# MAKING CLASSROOMS MORE AGE-FRIENDLY: STRATEGIES FOR INTERGENERATIONAL EXCHANGE

**DOI:** 10.1093/geroni/igac059.445

**Published:** 2022-12-20

**Authors:** Joann Montepare, Kimberly Farah, Lisa Borrero

## Abstract

Aging populations are reshaping how we think about teaching and learning in higher education. As a result, educational opportunities for intergenerational exchange are on the rise with the growth of the Age-Friendly University (AFU) initiative. Endorsed by GSA’s Academy for Gerontology in Higher Education, the AFU principles call for promoting intergenerational exchange to facilitate the reciprocal sharing of expertise between learners of all ages. Age diverse classrooms and learning spaces have distinctive needs and dynamics that instructors, and students, will need to learn how to navigate. This collaborative symposium, that brings together members of the Age-Friendly University (AFU) and Intergenerational Learning, Research, and Community Engagement (ILRCE) Interest Groups, will explore evidenced-based practices that contribute to successful experiences in and beyond the classroom. Montepare and Farah will provide an overview of the AFU initiative and intergenerational classroom issues. Jarrott and colleagues will address how technology and other strategies can prepare individuals for intergenerational learning experiences, including when circumstances limit in-person opportunities, such as during COVID. Dauenhauer and Heffernan will share insights from faculty about ways to develop and sustain a lifelong learning program that incorporates intergenerational interactions in the classroom experience. Leedahl will discuss strategies for developing age-friendly intergenerational internship experiences with community partners. Borrero, co-convener of the ILRCE Interest Group, will serve as the discussant.

